# Prioritizing programming to address the needs and risks of very young adolescents: a summary of findings across three humanitarian settings

**DOI:** 10.1186/s13031-017-0126-9

**Published:** 2017-11-14

**Authors:** Jennifer Schlecht, Catherine Lee, Brad Kerner, Meghan Greeley, Courtland Robinson

**Affiliations:** 1 Women’s Refugee Commission, 15 W 37th Street, 9th Floor, New York, NY 10018 USA; 2 Department of International Health, Johns Hopkins University, Bloomberg School of Public Health, 615 N. Wolfe Street, Baltimore, MD 21205 USA; 3 Save the Children US, Fairfield, CT USA; 4 Jhpiego, a Johns Hopkins University affiliate, 1615 Thames St., Baltimore, MD 21231 USA; 5 Present Address: International Medical Corps, Washington, DC USA

**Keywords:** Adolescents, Humanitarian, Refugee, Health, Sexual and reproductive health

## Abstract

**Background:**

Between 2013 and 2014, a series of qualitative and quantitative research efforts were undertaken in three conflict affected communities (Syrian refugees in the Bekaa Valley, Lebanon, Somali refugees in Kobe Refugee camp Ethiopia, and migrant communities from Myanmar settled in Tak Province, Thailand) that sought to understand the lived experiences of very young adolescents during emergencies. A research consortium that included the Women’s Refugee Commission, Johns Hopkins University, American University in Beirut, International Medical Corps in Ethiopia, Save the Children in Lebanon and the Adolescent Reproductive Health Network in Thailand convened around these activities.

**Methods:**

The core research initiative, “Sexual and Reproductive Health Needs and Risks of Very Young Adolescents in Humanitarian Contexts” involved three phases of research: 1) A program needs assessment completed with key informants working on adolescent sexual and reproductive health in each context 2) qualitative research, that incorporated participatory approaches, with adolescents 10–16 years of age, and adults in the community in all three settings, and 3) A household survey with 10–14 year-olds in Ethiopia and Thailand. An additional phase of research was added in Lebanon and Ethiopia, to further explore the issue of child marriage which was a prominent experience identified within the core research initiative on very young adolescents.

**Results:**

This research supplement, exploring the experiences during early adolescence, highlighted the crucial role of education, prominent experiences of insecurity, existing knowledge on body change and fertility, peer and family relationships among this age group, as well as concerns around child marriage (Lebanon and Ethiopia), child labor (Thailand), and gender roles.

**Conclusion:**

Given the findings presented within this supplement, there are broad implications for work with very young adolescents by humanitarian actors in the settings where research was undertaken, and potentially, more broadly. Programs that promote health and well-being during early adolescence are critically needed in settings of conflict and displacement, and coordination among the diverse range of actors involved in such programming will be essential. Additionally, SRH programs for adolescents (10–14 years of age) in emergencies should include, at minimum, sensitive care for survivors of sexual violence, menstrual hygiene management, life skills, and fertility education. Humanitarian programs can better engage with parents, teachers, and community leaders as partners in the development and implementation of programs. Finally, fundamental rights to protection and education for all adolescents must be upheld.

## Background

This paper summarizes findings from an article series compiled for a special *Conflict and Health* supplement on early adolescence in humanitarian contexts. It focuses on implications and related recommendations from a collection of research efforts that were undertaken among conflict affected communities from Myanmar, Syria and Somalia.

In humanitarian contexts, adolescents are rarely reached with effective health programming and interventions. Even when programs do exist, they tend to reach older youth rather than the full range of adolescents 10–19 years of age [1]. As a result, the diverse needs of this population, as they pass through critical development years, and the related opportunities to engage with young people at this time, are overlooked. UNICEF and others divide our understanding of adolescence into early adolescence (10–14 years of age) and late adolescence (15–19 years of age) due to the vast developmental differences observed across the span of these years [2]. During this period of change many will become married, sexually active, leave school, become orphaned, establish work outside the home, survive physical or sexual violence or become caretakers to their own children, parents, or siblings. It is during this decade of life that an intergenerational transfer of poverty often occurs, as poor adolescent girls give birth to impoverished children [2]. Limited access to education and restrictive gender roles during these early years may force young girls into situations of child marriage, domestic violence, and early childbearing that will shape their growth and potential for years to come [2]. Increasingly, literature points to the importance of interventions during early adolescence (10–14 years of age) in order to achieve good health, as well as protection from risks to health and well-being faced in later adolescence and adulthood [3]. Capacities developed, decisions made, and aspirations established during these years will have significant impacts on the long term sexual and reproductive health (SRH) and wellbeing of these individuals and ultimately their communities [3].

Dr. Robert Blum, a leading researcher on adolescent health, and colleagues have worked to develop a framework for understanding aspects that contribute to healthy growth and development in early adolescence. Based on existing literature, Blum, et al. [3] outlined five central goals for successful completion of early adolescence—“engagement with learning, emotional and physical safety, positive sense of self/self-efficacy, acquisition of life/decision-making skills, [and] physical and mental health.” When these goals are in place, they are associated with delayed sexual debut, improved contraceptive use, reduced transmission of sexually transmitted infections (STIs) and improved social, educational, and behavioral outcomes [3]. Although, globally, our understanding of this age group and the appropriateness of this proposed early adolescent framework is nascent, even less in known about how factors such as war, conflict and displacement impact the relevance and ability to achieve these goals.

This supplement presents a body of research that examines the experiences and needs of adolescents, and most prominently, those 10–14 years of age, herein referred to as very young adolescents (VYAs), displaced from Syria, Somalia and Myanmar. The supplement aims to document the lived experiences of VYAs, whose circumstances have been impacted by conflict and displacement, in order to improve their protection and inform immediate and longer-term humanitarian health response for this population. Many of the research activities presented through this supplement were informed by the Blum et al. framework, and sought to understand factors that may hinder or support the achievement of the central goals outlined in the framework. Settings for research activities were selected based on feasibility and partnerships, as well as the ability to maximize variation across sites, including geographic region, phase of the emergency (acute, chronic, and post-conflict), and type of settlement (camp, local settlement, and host community). This research was managed by the Women’s Refugee Commission, thanks to support from the Centers for Disease Control and Prevention (CDC) and dissemination support from the Government of Canada. Consortium partners included Johns Hopkins School of Public Health-Department of International Health; American University in Beirut, Department of Epidemiology; International Medical Corps in Ethiopia; Adolescent Reproductive Health Network in Thailand; and Save the Children in Lebanon.

Papers presented in this supplement include qualitative research conducted in three qualitatively different humanitarian settings: among Syrian refugees recently displaced in Lebanon, Somali refugees displaced in Ethiopia since 2011, and migrants from Myanmar in Thailand who have experienced long term displacement. Additionally, a paper summarizing findings from a household survey implemented with 10–14 year-olds from Somalia and Myanmar is presented, as well as supplemental research conducted on child marriage in Lebanon that followed the initial work on VYAs and was supported by NoVo Foundation. As a whole, these papers assist in developing a more complete picture and understanding of the experiences and concerns of VYAs, and particularly adolescent girls, during displacement due to conflict.

## Research methodology used in the supplement:

Four of the five research papers in this supplement [4–7] present research that was part of a global study on VYAs that employed three sequential phases of research and was informed by the Blum et al. conceptual framework. Each phase of research built upon knowledge gained from the prior. The first phase of research (program and needs assessment) involved key informant interviews with stakeholders working with adolescents in each country. Interviews explored possible sensitivities to the research topic, existing programs for adolescents in each setting, as well avenues to address future findings from the research.

The second phase of research (qualitative) was inductive in nature, and therefore researchers employed participatory methods to better understand the lived realities of VYAs as related to gender roles, safety, risks, community norms and expectations. Photo elicitation and community mapping were incorporated into focus group discussions conducted separately with boys and girls aged 10–12 years and 13–14 years. Focus group discussions were also held separately with those 15–16 years old, and adult community members (including parents of adolescents) to reflect on the experiences of VYAs.

Following the qualitative data collection, a quantitative household survey was then implemented among 10–14-year-olds in two of the three research settings (Thailand and Ethiopia). It was not possible to implement the household survey in Lebanon due to insecurity and other constraints. This third phase of research sought to quantify experiences and practices for VYAs through a deductive approach that gathered socio-demographic information, as well as data on: time use, school, parent/caregiver/adult connectedness, peer relationships, romantic relationship norms, puberty and SRH information and knowledge, safety and risk, personal and financial assets, early marriage (Ethiopia only) and child labor (Thailand only).

Following the implementation of the above efforts, an additional qualitative research effort was implemented among Syrian refugees in Lebanon and Somali refugees in Ethiopia, to specifically explore the issue of child marriage that had emerged within the VYA study. Findings from Lebanon are presented within this supplement.

## Discussion of supplement findings across contexts

Using mixed methods research, this supplement highlights a variety of factors that shape the experiences of VYAs, when conflict is a significant life event. In this section, we present key themes identified from the qualitative and quantitative research, including access to education, safety and insecurity, self-image and life skills, family and peer relationships, and child marriage (in Ethiopia and Lebanon) and child labor (in Thailand).

### Access to education

Across study sites and participants, education is described and understood to be critical for adolescent development, and is highly valued within each community. Aspirations expressed by VYAs are consistently linked to educational attainment. Access to learning environments, however, is highly varied.

Among Somali refugees in Ethiopia, VYAs have access to schools since displacement, which were not available in their own country during the conflict. As such, displacement has actually served to increase positive engagement in learning among VYAs in this setting [4]. In fact, enrollment is found to be extremely high (91.4%) among Somali 10–14-year-olds in the camp setting, where girls and boys appear to have equal access during these early years [5]. Qualitatively, barriers to regular school attendance for Somali VYAs in Ethiopia include distance to school, housework duties (for girls), lack of money, and marriage [4], with parental permission and health/ disability additionally identified as barriers within the quantitative study [5]. Domestic demands and early marriage appear to result in girls’ school enrollment declining, as they approach secondary school [4]. Unpublished data from the qualitative research in Ethiopia, finds that many VYAs from this setting are closely engaged with their mosque and imam—providing additional opportunities for VYAs to engage with informal learning networks that are positive for this age group. In community mapping VYA boys and girls identify schools and mosques as “safe” places, and teachers and imams as positive figures.

Similarly, among migrants from Myanmar in Thailand, school is highly valued by parents and adolescents. Although, qualitatively, adolescents report that access to formal education is not viewed as universal in camps or migrant communities [6], results from the quantitative data show that school enrollment among VYAs is relatively high, at 87%, and roughly equivalent between boys and girls [5]. Barriers to regular school attendance are identified through the survey as lack of money, work outside the home, and work inside the home [5], and FGDs suggest that the eldest daughters are most burdened with household responsibilities, leading to their later withdrawal from school [6]. VYAs interviewed in this setting identify teachers as a preferred source of health information, illuminating the trusting relationship at school that appears to exist in this context. Religious institutions are also highly valued and respected, suggesting an additional trusted source of information and socialization that overall strengthens the positive experiences of VYAs [6].

In Lebanon, Syrian refugees have experienced a great loss in both formal and informal education access. Prior to the conflict, parents and adolescents placed a high value on education, and adolescents saw education as the key to achieving their goals. However, currently in Lebanon, adolescents and adults do not view schools positively. For example, VYA boys describe being beaten and intimidated by Lebanese children, and girls express fears related to getting to and from school [7]. Transportation and enrollment costs are frequently prohibitive. Unlike in the other two sites, education access is largely limited, and few opportunities exist for engagement in any form of group learning, given the limited mobility that Syrian VYAs (most specifically girls) can realize in Lebanon [7].

### Safety and insecurity

Information was gathered on perceived physical safety across all three sites. Through a qualitative mapping activity, 10–14 year-olds described locations in their community where they feel safe and unsafe and then explained why. Across all sites, VYAs feel safer when in groups. VYAs do not wish to walk alone to school, by the roadside or in any more public space. Locations identified as secure are largely limited to home, youth centers, and public buildings used for official purposes. Examples of unsafe locations, are noted to be near water points, highways, outside settlements, anywhere after dark, and in some contexts—food distribution points [4, 6, 7]. Perceptions of safe locations are notably different between VYA girls and boys. Girls are far more likely to stay close with other girls in their peer group and clustered around the home or school, whereas boys describe more mobility and comfort in communal play areas. Fears around safety include physical harm and sexual violence (including rape and abuse) in all three sites, as well as kidnapping (Lebanon), arrest by the authorities and trafficking (Thailand) [4, 6, 7]. Among Syrians in Lebanon, insecurity causes parents and adolescents to worry more about the physical safety of adolescent girls which ultimately results in their limited mobility. Brothers and male relations are described as taking on a role protective of girls, as parents might be preoccupied with basic economic concerns [7].

Through the household survey, both Somali VYAs in Ethiopia (11%) and VYAs from Myanmar in Thailand (25%), cite safety concerns as reasons for missing or not attending school [5]. An additional 46% of VYAs in Myanmar and 11% of VYAs from Somalia identify “work” (distinct from household work) as a reason for not attending school, presenting possible safety/protection implications [5]. Though not all data are presented in this issue, unpublished survey results show that, while the top three risks identified by adolescents in both sites are poverty, being forced to work, and the inability to attend school regularly, fear of physical violence is cited by roughly 34% of Somali VYAs and 28% of VYAs from Myanmar, with the highest group citing this risk being Somali girls (39%).

### Self-image and life skills

Across all sites, VYAs are consistently viewed as “children” by their communities and peers. Puberty and marriage are identified as life events that typically shift roles and community expectations. Such transitions are however understood by VYAs differently in each context, providing us with insight into their self-image and self-concept as boys and girls.

The majority of adolescents from Myanmar have been displaced for more than 5 years, meaning that they are unlikely to have many memories of their communities of origin [5]. Parents express the impression and fear that adolescents are disconnected from their culture and values. Although some VYAs express ambitions of higher education, their underlying narrative is primarily one of hopelessness, stress, and fear related to trafficking and arrest by immigration police [6]. Adolescents report that most become sexually active during the transition from early to later adolescence, but most possess very little SRH knowledge [6]. About 80% of VYA boys say they feel comfortable with pubertal changes, compared to 65% of VYA girls [5]. A surprisingly low number of VYAs who report starting puberty say that they learned about these changes before they occurred (1 in 3 girls and 1 in 5 boys), with fewer than half stating that they had the information they needed to understand what was happening with their bodies [5]. The majority of girls who began menstruating (76.7%) also reported access to all basic menstrual hygiene supplies (soap, water, private washing facilities and locally appropriate pads or cloth) to support their ongoing engagement in society during menstruation [5]. Adolescents of all ages note that pregnancy is highly stigmatized, but not uncommon among young girls in their community; when girls become pregnant, they risk full isolation by their peers and many are known to seek unsafe abortion [6].

Syrian adults and adolescents alike talk about how displacement to Lebanon forces children to grow up too quickly. Some parents express worry about their children being exposed to the more liberal gender norms of Lebanese society. As a result, mobility and social interactions are largely restricted for girls, resulting in limited peer interactions and engagement in programs. Girls express a significant loss of hope for their future since displacement, related to their loss of mobility and opportunity. Limited access to education opportunities impacts future aspirations for both boys and girls. Even when education is accessible, many Syrian adolescents describe that they experience derogatory comments and risk violence from Lebanese youth, which discouraged many from further attendance. Both boys and girls express a loss of hope in their future, which may contribute to a declining self-image. Psychological health is broadly defined as poor- and adolescents express feeling overwhelmed by the stress of the conflict and displacement. An adolescents’ reputation, is closely linked to self-image—a notion especially valued, and viewed as precarious in these new unfamiliar settings, particularly by adolescent girls. Knowledge of puberty appears limited among adolescents of both sexes, and many questions about body change were met with laughter and shyness. Adolescents share that any SRH information they have, is primarily from phones or television and both parents and adolescents express mutual discomfort discussing these topics [7].

In Kobe camp in Ethiopia, about 80 and 96% of VYA boys and girls respectively, from Somalia say they feel comfortable with pubertal changes [5]. Adolescents at all ages demonstrate relatively high comfort levels in discussing body change during puberty—including menstruation, hair growth, breast development and voice change [4]. Of those who have been through puberty, nearly all Somali girls (95%) and the majority of boys (69%) reported that they had learned of puberty before it occurred [5]. However, very few girls in this setting reported access to basic menstrual hygiene management resources explored within this survey (soap & water, private washing facility, and locally appropriate sanitary pads or cloth) [5]. Gender roles, in this community, tend to be highly differentiated with the boys allowed more freedom outside the house, including the ability to attend school, whereas girls are perceived to be “better suited for the house tasks than boys” [4]. Marriage is accepted by girls as a critical step in their development, and thus understood to be a goal after puberty. Boys and girls (if not related) may play together till around 10 years old, after which interactions are more restricted.

### Family and peer relationships

Somali adults and VYAs agree that while some roles of boys and girls are changing in the camp, “there are deep cultural traditions that persist” [4]. The household surveys show that about 40% of Somali VYAs feel it is OK to talk or spend time with peers of the opposite sex, though only 30% feel it is OK for boys and girls to spend time alone. Although VYA girls and boys rarely, if ever, move around the camp alone, same-sex peers are largely part of their educational landscape. Somali adolescents of all ages identify parents and family networks as the key source of support and information on sensitive topics related to body change and puberty. Families are described as tightly knit, and adolescents express trust and confidence in the guidance and support provided by their parents. Girls and boys seem to acknowledge the role of the father as the decision maker in the household, especially on issues of education and marriage. In terms of information about SRH and health, about 75% of Somali VYA girls get information about puberty from their mothers and even more (92%) get information from their mothers on pregnancy [5]. Parental connectedness is relatively high, and about 90% of VYA adolescents report feeling that their parents care for them, and 88% that their parents listen [5].

Adults and adolescents from Syria shared that peer interactions are significantly limited since displacement. Parents report that they respond to the less familiar environment and fears for their children, by restricting mobility and social interaction. This shift is especially impactful for girls, who express deep sadness at the isolation they now face. This is especially challenging for Syrian girls who have come from more progressive areas of Syria. Syrians in Lebanon note two key changes in family relations brought on by displacement and the hardships that resulted. One is that traditional boundaries of privacy and personal modesty are challenged as families must share often cramped quarters and children are exposed to marital relations that they might not have been, back in Syria. The second, somewhat contradictory to the first, is that parents describe spending less time with their children: “Before the crisis, the parents used to be more involved with their kids’ lives but now the father has many other things to worry about and the mother always worries how she will be able to provide for her kids” [7].

In Thailand, VYAs from Myanmar describe high dependence on peers for support. Both adolescents and adults agree that most 10–14-year-olds spend the majority of their day with peers, either at school or in recreational spaces (though some noted the lack of safe spaces outside of school and home) [6]. Results from the household surveys show that about 32% of VYAs from Myanmar feel it is OK to talk or spend time with friends of the opposite sex, and 23% feel it is OK to spend time alone with these friends. Families and parents are considered caring and supportive, but information sharing (especially around puberty and body change) is less frequently a component of the relationship, than seen in the Somali site. Only 47% of girls get information about puberty and 57% get information on pregnancy from their mothers. Even fewer boys, 23%, get information on puberty from their fathers (with 15% getting this information from teachers instead) and 36% learn about pregnancy from their fathers compared to 71% who get this information from teachers. About 90% of VYAs feel their parents care, and 72% feel their parents listen [5].

### Child marriage and child labor

Among Somalis in Ethiopia, early marriage is a widespread concern among VYA, especially young girls, who understand the community expectations to marry at a young age will likely lead to an end of her education [4]. Boys, on the other hand, feel supported to pursue their education and personal goals. Among Syrians in Lebanon, both children and parents express a view that conflict and displacement have changed the customs around marriage – expectations for marriage are lowered, ceremonies have become less expensive and easier to arrange, and pressures to marry adolescent girls are increasingly for reasons of protection or economics [7, 8]. Some respondents speak of a new awareness of the negative effects of early marriage, resulting from programs implemented in their setting, and believe that families should resist pressures to marry their girls at young ages [4, 7].

Among populations from Myanmar displaced in Thailand, concerns about early marriage are not expressed; instead, adolescents and adults worry about the pressures on children to work in order to help the family survive (this is true especially among the migrant and displaced populations living outside of refugee camps). Concerns about child labor are compounded by worries that adolescents could be trafficked into exploitative work conditions and that they could face mistreatment both by employers and local authorities [6].

### Implications for programs

The period from 10 to 14 years of age, is a time of significant biological, physical, cognitive and emotional change. Research has connected positive engagement in learning, emotional and physical safety, positive sense of self and life skills during these years with improved SRH outcomes [3]. Additionally, more recent research (largely from North America and Western Europe) has concluded that peers and parents largely shape the gender norms embraced during early adolescents which significantly impact SRH outcomes [9]. The establishment of programs to reach VYAs in humanitarian contexts is complex and will present challenges reflective of significant resource constraints already experienced by families, and the limited mobility frequently faced by adolescents, and adolescent girls, specifically, among others. Given the findings from this collection of research, among displaced populations from Syria, Somalia and Myanmar, there are broad implications for work with adolescents by humanitarian actors in these specific humanitarian settings, and potentially, more broadly.

#### Programs that promote health and well-being during early adolescence are critically needed in settings of conflict and displacement

The years of 10–19 encompass years of significant change and opportunity. In humanitarian crises, adolescents face immense risks to their long-term health and well-being—enduring increased risks to negative life events compounded by reduced engagement in protective and supportive programming. In humanitarian contexts, it is critical that steps be taken to protect and support the emotional well-being and developing sense of self of adolescents, and interventions for VYAs offer an important opportunity [10]. Inaction to provide sufficient protection and supports can have long-term detrimental outcomes. Programs for VYAs serve to provide critical information, skills or resources to adolescents, before they enter their highest risk years—when they are most at risk for child marriage, disenrollment from school, unplanned pregnancy, and transmission of sexually transmitted illnesses among others. Within this supplement, research is presented in which VYAs express concerns about these issues- including safety, protection and their access to services (including education). Research from development contexts has suggested that VYA programs should promote emotional and physical safety, engagement in learning, positive sense of self and life skills. Activities that achieve these broad objectives can be embraced by relevant sectors/clusters and adapted to humanitarian contexts.

#### SRH programs for adolescents (10–14 years of age) in emergencies should include, at minimum, care for survivors of sexual violence, menstrual hygiene management, life skills, and fertility education

During a humanitarian emergency, VYAs face risks to their sexual health from a very early age, and must have access to the Minimum Initial Service Package for Reproductive Health as a life-saving intervention from the earliest phase of an emergency [10]. This will most critically include care for survivors of sexual violence and menstrual hygiene supplies. This research highlighted the critical gap in supplies for menstrual hygiene management (MHM), especially among Somali refugees in Ethiopia, as well as significant fears of sexual violence. Prior research efforts have acknowledged a gap in SRH care for adolescents and a gap in care for survivors of sexual violence [11]. Additionally, young people need preparation for their sexual lives, before they become sexually active [12]. Interviews conducted with conflict affected communities from Syria, Somalia and Myanmar (adolescents, parents and community leaders) underscored that puberty and fertility education is widely accepted as appropriate for this age group. Education about body change and fertility can be undertaken during humanitarian response’s transition to comprehensive SRH, and in collaboration with communities (families, teachers and leaders). There are additionally, a number of girls and boys puberty books that have been developed and could be adapted to a variety of contexts [13]. UNESCOs Comprehensive Sexuality Education Curriculum provides a basis for school and non-school based education efforts [12].

#### Humanitarian programs can engage with parents, teachers, and community leaders as partners

There are many natural partners when seeking to educate VYAs about their bodies. In this research, parents, teachers and community/ religious leaders were identified as important, trusted sources of information by adolescents, and many expressed interest in possessing more accurate information that they could share with VYAs. Approaches to improve SRH during early adolescence will need to move beyond facility-based care and reach VYAs through existing information channels. VYAs are largely invisible to the existing health systems- having moved beyond routine childhood visits, and yet to embark on their reproductive years. As such programs should be designed to reach VYAs where they currently congregate—in locations such as schools, religious institutions, communal areas—or through current, trusted information sources (mothers, fathers, sisters, brothers, teachers, etc). Supporting previously existing support networks and resources are particularly important in a humanitarian crisis, where these structures may have been compromised during conflict or displacement. Although VYA programs contribute to improved SRH outcomes, programmers can also work carefully with community members to determine terminology adopted for SRH-related interventions targeting this age group. “SRH” is unlikely to feel descriptive of the actual planned intervention, nor acceptable to the local community.

#### The right to education must be upheld by actors now engaged across humanitarian action

Education, of both boys and girls, is a fundamental human right and key to achieving key health outcomes for individuals and communities. However, in many settings of conflict and displacement, access is severely limited due to concerns around safety, inequitable gender norms, capacity of education systems to absorb new populations, and poverty. Education plays an enormous role in achieving key health outcomes for individuals and communities. In crises, actors should support and build on the intrinsic value placed on education by adolescents and adults alike, by ensuring that all adolescents have safe access to education during these critical years of development.

#### The protection of adolescents from all forms of violence is paramount

Research presented within this supplement highlights the reality that many adolescents continue to be exposed to violence, and are preoccupied by related fears even after fleeing conflict. Current protection mechanisms appear insufficient for the diversity of protection issues raised across settings where research was undertaken. Girls express fears of sexual violence and abuse when undertaking routine activities- including moving to school, going to markets and fetching water. Issues of child marriage (Lebanon and Ethiopia), trafficking (Thailand), and abduction (Lebanon) were prominent. Despite international guidance on child protection and protection from gender based violence, implementation is yet to ensure the safety of young people from violence.

#### Coordination is needed among health, education and protection actors, working to address the needs of adolescents in a humanitarian crisis

SRH needs during early adolescence are rarely “facility-based” or falling within traditional health service delivery models. For the humanitarian system to effectively achieve goals during adolescence, that will promote their long-term well-being and health (emotional and physical safety, engagement in learning, positive sense of self and life skills) coordination and communication is required across clusters and multiple actors. Establishing a structured communication mechanism among actors engaged in programming that may benefit this population would provide needed mechanisms for coordination, advocacy and the measurement of successes.

## Conclusions

As noted at the outset of this paper, research on SRH of refugee and migrant populations, while growing, remains a neglected field [14]. While reproductive health became an area of increasing concern in the mid-1990s, research continued to be limited by the view that reproductive health is a long-term issue, and thus less critical to understand and address during humanitarian emergencies than more immediate concerns [15]. Fortunately, the humanitarian field is becoming increasingly aware of the critical need for SRH services in complex emergencies and disasters. Still, the SRH needs of adolescents, and particularly VYAs remain largely overlooked. The findings of the qualitative and quantitative reseearch undertaken as part of this supplement suggest that conflict and displacement disrupt the social fabric of family and community and interrupts the physical, emotional, social, and cognitive development of VYAs. The disruptions in living conditions, family relationships, school attendance, and cultural practices---coupled with the new uncertainties and insecurities brought about by displacement into foreign countries and cultures—increase the physical, sexual, and psychological risks facing already vulnerable VYA refugees, migrants, and displaced persons. New approaches are needed to focus resources on both the developmental and health needs of this population as they come of age in contexts of conflict and displacement.
